# DeDiAttack: Enhancing Transferability of Unrestricted Adversarial Examples via Deformation-Constrained Diffusion

**DOI:** 10.3390/s26092823

**Published:** 2026-05-01

**Authors:** Bin Qu, Anjie Peng, Shijie Zhao

**Affiliations:** 1 Jianghuai Advanced Technology Center, Hefei 230037, China; penganjie@swust.edu.cn (A.P.); shijiezhao18@163.com (S.Z.); 2 School of Computer Science and Technology, Southwest University of Science and Technology, Mianyang 621010, China

**Keywords:** unrestricted adversarial attacks, black-box transferability, visual naturalness, deformation-constrained, diffusion models

## Abstract

DNNs are highly vulnerable to adversarial examples (AEs). To achieve high transferability, traditional AEs often introduce unnatural artifacts that are easily perceptible to the human eye. Unrestricted attacks have emerged as a promising paradigm to generate more natural unrestricted adversarial examples (UAEs). However, existing UAEs struggle to balance visual fidelity and black-box transferability. Color-based attacks produce noticeable unnatural visual mutations, and diffusion-based attacks transfer poorly to unknown black-box models. We observe that directly injecting unconstrained random perturbations into the diffusion latent space destroys the normal distribution of data, thereby causing a distribution shift. Distribution shifts degrade adversarial perturbations into invalid noise and cause surrogate model overfitting. Furthermore, introducing elastic deformation during the denoising process forces surrogate models to focus on highly transferable features. As a result, we propose an unrestricted attack based on deformation-constrained diffusion, called DeDiAttack. Our method utilizes the manifold prior knowledge of diffusion models to translate elastic deformations into smooth fluid changes. The mechanism effectively eliminates unnatural artifacts and generates highly natural and transferable UAEs. Extensive black-box experiments demonstrate that DeDiAttack outperforms existing attacks and improves the black-box transferability of generated UAEs by 7.2% on the ViT-B surrogate model. The proposed method also provides a useful robustness evaluation tool for vision-based sensing and imaging systems.

## 1. Introduction

Deep neural networks (DNNs) exhibit remarkable performance across various visual tasks (e.g., image classification [1], object detection [2], and semantic segmentation [3]). However, extensive studies [4,5] have demonstrated the high vulnerability of DNNs to adversarial examples (AEs). Attackers mislead models into making incorrect predictions by adding visually imperceptible perturbations to clean images. Because AEs exhibit cross-model transferability [6,7], they pose a severe security threat to real-world systems. This issue is particularly important for sensing and imaging systems, where vision-based sensors, image signal-processing pipelines, and downstream recognition models are often deployed in safety- or mission-critical environments. Therefore, understanding the transferability and naturalness of adversarial examples is valuable for evaluating the robustness of practical sensing and imaging applications.

Traditional adversarial attacks [4,5] restrict the perturbation magnitude within a specific $L_{p}$-norm ball (e.g., ϵ≤16/255) in the RGB space to ensure visual imperceptibility. Researchers have introduced various optimization methods (e.g., momentum optimization [8,9] and input transformations [10,11]) to boost the transferability of AEs under black-box settings. However, updating perturbations based on pixel-level gradients often generates high-frequency noise. Differences in pixel-level features significantly reduce attack performance when the target and surrogate models belong to completely different network architectures (e.g., transferring from CNNs to Transformers). Additionally, high-frequency noise added to AEs destroys the content structure of images, making the AEs easily perceptible to the human eye.

**Figure 1:**
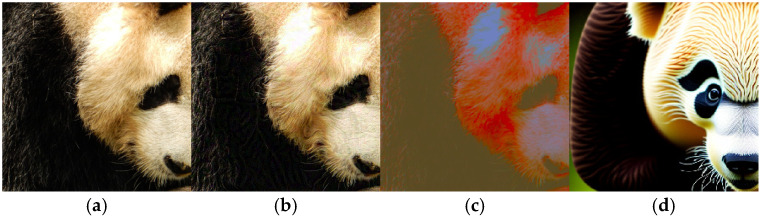
Visual comparison of AEs/UAEs generated by different attacks. It is evident that AEs generated by GI-FGSM [9] exhibit significant pixel artifacts, UAEs generated by NCF [15] show obvious color distortions, and UAEs generated by ACA [17] exhibit visible structural deformations. (**a**). Clean (**b**). GI-FGSM [9] (**c**) NCF [15] (**d**). ACA [17].

Recent works [12,13] have explored unrestricted attacks to overcome the limitations of traditional $L_{p}$-norm constraints. Unrestricted attacks deceive DNNs by modifying image colors [14,15] and textures [16] or by utilizing generative models [17,18] to craft more natural unrestricted adversarial examples (UAEs). Although the generated UAEs maintain high visual naturalness, UAEs typically lag behind traditional attacks in black-box transferability. When facing unknown black-box models, existing unrestricted attacks [14,15] often focus on global semantic or color modifications, limiting the attack performance of the generated UAEs. As illustrated by the visual results in Figure 1, AEs/UAEs crafted by transfer and color-based unrestricted attacks are easily perceived by the human eye. Improving UAE transferability across different models while maintaining naturalness remains challenging.

Diffusion models (DMs) [19,20] exhibit powerful generative and implicit discriminative capabilities by learning massive data distributions. Recent studies, such as Latent Transfer Attack (LTA) [21], Improving Adversarial Transferability and Imperceptibility with Loss Landscape and Diffusion Model [22], and NatADiff [23], further indicate that latent-space adversarial optimization has become an important trend for jointly improving transferability and visual naturalness. On the one hand, the latent space of DMs contains rich semantic priors and guarantees the naturalness of generated images through the denoising process [24]. On the other hand, spatial elastic deformation simulates diverse local deformations, forcing surrogate models to focus on more transferable features [25]. We observe that injecting unconstrained random perturbations directly into the latent space of DMs easily causes the latent representations to deviate from the prior normal distribution of natural images. This deviation causes the perturbation to degrade into invalid noise during the denoising process and overfit the surrogate model. Therefore, introducing elastic deformation during the denoising process enriches local adversarial details. The continuous manifold properties of DMs translate the applied elastic deformations into smooth fluid changes to generate more natural and highly aggressive UAEs. Furthermore, forcing the perturbation distribution in the latent space to approximate the standard normal distribution can effectively eliminate invalid noise that causes model overfitting.

According to the above insights, we propose an unrestricted attack based on deformation-constrained diffusion, called DeDiAttack. To avoid unnatural artifacts, we first invert the clean image into the latent space of a Stable Diffusion Model (SDM) [26] for adversarial optimization, rather than operating directly in the pixel space. We then introduce a spatial elastic deformation mechanism during the decoding stage of the iterative optimization to enrich local image details. Unlike existing unrestricted attacks that mainly emphasize latent optimization or adversarial sampling, we introduce a deformation-based input transformation to the diffusion latent space and exploit the diffusion manifold to convert the deformation into smooth natural visual changes. DeDiAttack leverages the rich manifold prior knowledge of DMs to translate the applied elastic deformations into smooth fluid changes. Furthermore, we design a distribution alignment mechanism to force the distribution of the adversarial latent representation to approximate the standard normal distribution. The distribution alignment mechanism prevents the injected perturbations from causing overfitting and degrading into invalid noise. Finally, we align the self-attention maps during the denoising process to maintain the image quality of the generated UAEs. The ultimately generated UAEs maintain visual naturalness and exhibit outstanding transferability against unknown black-box models. This capability is also valuable for robustness evaluation in vision-based sensing systems, where highly natural and transferable adversarial examples can provide more realistic stress tests for sensor data processing and downstream recognition modules.

We summarize our main contributions as follows:

- We propose an unrestricted attack based on deformation-constrained diffusion, called DeDiAttack. Our method utilizes the manifold prior knowledge of DMs to translate elastic deformations into smooth fluid changes, thereby generating natural and highly transferable UAEs.
- To improve UAE transferability, we design a distribution alignment mechanism that eliminates invalid noise that causes surrogate model overfitting. To maintain the UAE image quality, we introduce a self-attention map alignment mechanism to ensure image structure integrity.
- Extensive experiments on various model structures and defense approaches demonstrate the superiority of DeDiAttack over existing unrestricted attacks; for instance, our method improves the ASR by 7.2% when using ViT-B as the surrogate model.

## 2. Related Works

### 2.1. Diffusion Models

DMs are based on stochastic processes and Markov chains [19,20]. The forward process converts the original image into pure noise by gradually adding Gaussian noise. The reverse denoising process utilizes neural networks to iteratively recover the original data distribution from Gaussian noise. Benefiting from pre-training on large-scale datasets, DMs exhibit the powerful ability to generate high-quality images [24]. DMs are widely adopted for various generative visual tasks (e.g., image inpainting [27], image-to-image translation [28], and local image editing [29]). Recent works [17,18] have explored the potential of DMs in adversarial attacks. DMs possess rich manifold prior knowledge, which can translate the perturbations applied to images into smooth fluid changes. Continuous manifold properties effectively avoid the generation of unnatural deformations that violate physical principles.

### 2.2. Adversarial Attacks

Traditional adversarial attacks [4,5] deceive deep neural networks by applying imperceptible perturbations to input images. The perturbation magnitude is strictly restricted within a specific $L_{p}$-norm ball (e.g., ϵ≤16/255) to ensure the visual imperceptibility of AEs. For instance, FGSM [4] generates perturbations by calculating the single-step gradient of the loss function. PGD [5] generates perturbations through multi-step iterative optimization. Traditional attacks achieve remarkably high success rates under white-box settings with known model structures. However, $L_{p}$-norm-constrained perturbations are often not transferable to unknown target models. Traditional AEs easily become ineffective when facing black-box models with unknown structures or systems deployed with defense mechanisms.

### 2.3. Transfer Attacks

Transfer attacks [6,7] aim to successfully deceive unknown target models using AEs generated from white-box surrogate models. Researchers have proposed various methods to improve AE transferability, including gradient-based [8,9], input transformation-based [10,11], advanced-objective [30], model-related [31], and ensemble-based [32] attacks. In gradient-based attacks, MI-FGSM [8] avoids falling into local optima by accumulating historical gradient directions during iterations. GI-FGSM [9] utilizes global momentum initialization to improve update direction stability. In input transformation-based attacks, Admix [10] calculates more generalizable gradients by mixing the original input image with scaled versions. BSR [11] enriches adversarial details by randomly shuffling and rotating image blocks of the input image. Recent studies have further demonstrated the effectiveness of input transformation from complementary perspectives [33,34]. Mixed-frequency inputs improve transferability by jointly exploiting low- and high-frequency components, while dynamic transformation learning adaptively learns transformations that produce more generalizable adversarial gradients. These findings further support our motivation for introducing deformation-based transformation into the diffusion latent space rather than directly perturbing pixels. Introducing momentum enhancement or input transformation methods inevitably destroys the original structure of images, with this severe image degradation making the resulting AEs easily perceptible to the human eye.

### 2.4. Unrestricted Attacks

Unrestricted attacks [12,13] abandon traditional $L_{p}$-norm constraints. These attacks generate more natural UAEs by modifying the semantic information of images (e.g., colors [14,15] and latent representations [17,18]). Color-based attacks (e.g., cAdv [14] and NCF [15]) deceive unknown models primarily by altering image color distribution. DM-based attacks combine the perturbation optimization process with the latent space. ACA [17] generates more diverse and natural UAEs through an aligned low-dimensional manifold. DiffAttack [18] optimizes the adversarial loss during the reverse denoising process. Unrestricted attacks show unprecedented capabilities in improving visual quality. LTA optimizes perturbations in generative latent spaces to improve cross-architecture transferability [21]. Liu et al. combined DDIM inversion with loss-landscape flattening to enhance both transferability and imperceptibility [22]. NatADiff guides the diffusion trajectory toward natural adversarial samples with improved image quality and black-box transferability [23]. However, existing unrestricted attacks exhibit low transferability when facing unknown black-box models. The generated UAEs struggle to balance visual fidelity and black-box transferability. From the defense perspective, FlowPure further shows that purification-based generative defenses are becoming stronger, highlighting the necessity of evaluating unrestricted attacks under more challenging defense settings [35]. AdvDiff [36] performs adversarial sampling using target classifier gradients. VENOM [37] utilizes text prompts as driving guidance.

## 3. Methodology

### 3.1. Problem Formulation

For a clean image x with a true label y, we define a deep neural network classifier parameterized by θ as $F_{\theta }$. Unrestricted adversarial attacks aim to generate visually imperceptible perturbations for the input image x to construct a UAE $x^{\prime }$. The UAE $x^{\prime }$ misleads the classifier into outputting incorrect predictions. We formulate the unrestricted attack objective as Equation (1):

(1) $$F_{\theta }\left(x^{\prime }\right)\neq y\;s.t.\;\;x^{\prime }\;\text{is looking-natural.}$$

We mainly focus on the black-box attack scenario in this research. Attackers cannot access the parameters and internal structure information of the target model under black-box settings. Therefore, we exclusively use an accessible surrogate model $F_{\theta }$ to craft UAEs and rely on their cross-model transferability to deceive the target model. Our proposed method is as described in Algorithm 1.
**Algorithm 1.** The optimization procedure of DeDiAttackInput:
  Clean image x

  Ground-truth label y

  Surrogate classifier $F_{\theta }$

  Stable Diffusion Model SDM

  Number of optimization iterations N

  Number of deformation samplings K
Output: 
  Unrestricted adversarial example $x^{\prime }$
1: $z^{\prime }$ <- DDIM Inversion (x)
2: Initialize the adversarial latent variable with $z^{\prime }$
3: for i=1 to N do
4:   $\hat{x}$ <- Decode ($z^{\prime }$)
5:   for k = 1 to K do
6:     Sample a random deformation noise map $n_{k}$
7:     Update $n_{k}$ according to the classification loss in Equation (4)
8:     $x_{k}$ <- T ($\hat{x}$, $n_{k}$)
9:   end for
10:    Compute the classification loss $L_{cls}$ using Equation (6) and $F_{\theta }$
11:    Compute the distribution alignment loss $L_{kl}$ using Equations (7) and (8)
12:    Compute the structure preservation loss $L_{struct}$ using Equation (9)
13:    Update $z^{\prime }$ by AdamW according to the total objective in Equation (10)
14: end for
15: $x^{\prime }$ <- Decode ($z^{\prime }$)
16: return $x^{\prime }$


### 3.2. Overall Framework

**Figure 2:**
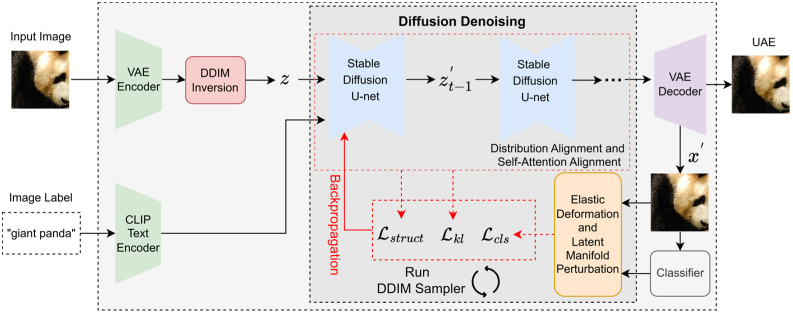
The overall framework of the proposed DeDiAttack. Our method directly optimizes an adversarial latent representation $z^{\prime }$ within the latent space of SDM. During denoising, an elastic deformation mechanism and three specific loss functions are employed to generate highly transferable and visually natural UAEs.

Figure 2 illustrates the overall framework of the proposed method. DeDiAttack aims to generate highly transferable and visually natural UAEs. We utilize DDIM inversion to map the clean image x to an initial latent representation z. We directly optimize an adversarial latent representation $z^{\prime }$ in the latent space of an SDM. Optimizing within the latent space avoids unnatural artifacts caused by direct image manipulation in the pixel space. We decode the adversarial latent representation $z^{\prime }$ into an image $x^{\prime }$ at each iteration of the optimization. We subsequently apply an elastic deformation mechanism to the decoded image $x^{\prime }$. The manifold prior knowledge of the SDM translates the elastic deformation into smooth fluid changes. The continuous manifold properties avoid generating rigid pixel distortions that violate physical common sense.

We design three losses during the optimization process. The classification loss $L_{cls}$ is used to deceive the classifier. The alignment loss $L_{kl}$ is utilized to improve the transferability of the generated UAEs. The structural loss $L_{struct}$ is employed to maintain image quality.

### 3.3. Elastic Deformation

We introduce an elastic deformation mechanism to perform input augmentation on the decoded image $x^{\prime }$. The elastic deformation simulates diverse and subtle spatial deformations. We first sample a random noise map ξ from a uniform distribution. We extract the original control point coordinate set O of the image. We adjust the original control points using the random noise map ξ to generate a deformed point set P as shown in Equation (2):

(2) P=O+ξ.

Then, we apply a thin-plate spline interpolation algorithm to process the deformed control points. The thin-plate spline interpolation algorithm calculates a mapping function for coordinate offsets to generate a distorted spatial grid. Finally, we resample the pixels of the decoded image $x^{\prime }$ according to the distorted spatial grid to generate an augmented image $\hat{x}$ with elastic deformation according to Equation (3):

(3) $$\hat{x}=T\left(x^{\prime },P\right),$$

where T represents the comprehensive deformation operation of thin-plate spline interpolation and grid sampling based on the deformed point set P.

### 3.4. Latent Manifold Perturbation

Unconstrained elastic deformation easily produces excessive distortions in the generated UAEs. The initial noise map ξ requires adaptive adjustment based on the feedback of the surrogate model $F_{\theta }$. This adaptive adjustment limits UAE deformation to a reasonable range. The cross-entropy loss $L_{ce}$ is used to measure the prediction error of the surrogate model $F_{\theta }$ on the augmented image $\hat{x}$ with respect to the true label. Updating along the negative gradient direction of the cross-entropy loss $L_{ce}$ determines the deformation direction with minimal image quality loss. A single-step update operation generates an optimized deformation noise map $\hat{\xi }$ as formulated in Equation (4):

(4) $$\hat{\xi }=\xi -\rho \nabla_{\xi }L_{ce}\left(F_{\theta }\left(\hat{x}\right),y\right),$$

where ρ represents the update step size and $\nabla_{\xi }$ denotes the gradient calculation regarding the noise map.

After obtaining the optimized deformation noise map $\hat{\xi }$, we represent the final deformed point set $\hat{P}$ as Equation (5):

(5) $$\hat{P}=O+\hat{\xi }.$$

We use the final deformed point set $\hat{P}$ to generate the final deformed image. To deceive the classifier, the surrogate model $F_{\theta }$ computes the average cross-entropy loss across N groups of different deformed images as expressed in Equation (6):

(6) $$\arg\underset{z^{\prime }}{min}L_{cls}=-\frac{1}{N}\sum_{i=1}^{N}L_{ce}\left(F_{\theta }\left(T\left(x^{\prime },{\hat{P}}_{i}\right)\right),y\right),$$

where N indicates the total number of deformation samplings and ${\hat{P}}_{i}$ represents the i-th deformed point set.

### 3.5. Distribution Alignment

The data in the latent space of DMs follows a standard normal distribution. Unrestricted adversarial perturbations usually inject unconstrained random perturbations into the latent space. The injected perturbations destroy the normal distribution of data, causing distribution shifts, which degrade adversarial perturbations into invalid noise during the denoising process. Furthermore, the invalid noise easily causes UAEs to overfit the surrogate model $F_{\theta }$, reducing the transferability to unknown models.

We introduce a distribution alignment mechanism to force the added noise to fit the manifold space of natural images. The distribution alignment mechanism improves the transferability of generated UAEs by eliminating invalid noise. We first calculate the perturbation difference δ between the adversarial latent representation $z^{\prime }$ and the initial latent representation z as shown in Equation (7):

(7) $$\delta =z^{\prime }-z.$$

Then, we extract the mean $\mu_{\delta }$ and variance $\sigma_{\delta }^{2}$ of the perturbation difference δ in the current computational graph. We force the perturbation distribution to strictly approximate the standard normal distribution N(0,I) according to Equation (8).

(8) $$\arg\underset{z^{\prime }}{min}L_{kl}=-0.5\sum \left(1+\ln\left(\sigma_{\delta }^{2}\right)-\mu_{\delta }^{2}-\sigma_{\delta }^{2}\right).$$

### 3.6. Structure Preservation

Adversarial optimization in the latent space inevitably changes the content structure of the generated images. Research by VENOM [37] has shown that self-attention maps naturally capture the internal structural information of images during the diffusion denoising process.

Inspired by DiffAttack [18], we utilize the self-attention maps of DMs to preserve the content structure of generated UAEs. We calculate the self-attention maps of the initial latent representation z and the adversarial latent representation $z^{\prime }$ during the denoising steps. We denote these maps as A and $A^{\prime }$, respectively. We force $A^{\prime }$ to approach A through the structural loss formulated in Equation (9):

(9) $$\arg\underset{z^{\prime }}{min}L_{struct}={\left||A^{\prime }-A|\right|}_{2}^{2}.$$

The final objective function $L_{total}$ is shown in Equation (10).

(10) $$\arg\underset{z^{\prime }}{min}L_{total}=\alpha L_{cls}+\beta L_{kl}+\gamma L_{struct},$$

where α, β, and γ are the hyperparameters used to balance the weights of the loss terms.

## 4. Experiments

### 4.1. Setup

**Datasets**. Consistent with previous studies [17,18], experiments are conducted on the ImageNet-compatible dataset [38]. The dataset contains 1000 images with a resolution of 299×299, covering rich category information. During the experiments, all images are uniformly resized to 224×224.

**Models**. We select eight mainstream deep learning models for experiments to verify attack effectiveness, including CNN-based models, such as Res-50 [39], VGG-19 [40], and Mob-v2 [41], and Transformer-based models, such as ViT-B [42], Swin-B [43], and DeiT-B [44]. In addition, we introduce various defense approaches to assess attack robustness, including HGD [45], R&P [46], JPEG [47], Bit-Red [48], and DiffPure [49].

**Attacks**. We compare DeDiAttack with nine existing attacks. Restricted transfer attacks include MI-FGSM [8], GI-FGSM [9], and Admix [10], where Admix is an input transformation-based attack. Unrestricted attacks include cAdv [14], NCF [15], ACA [17], DiffAttack [18], AdvDiff [36], and VENOM [37]. The latter four attacks, along with our method, are DM-based attacks. For the evaluated restricted transfer attacks, the maximum perturbation magnitude is strictly set to ϵ=16/255.

**Evaluation Metrics**. We use the attack success rate (ASR) to measure attack effectiveness. ASR is the proportion of generated AEs/UAEs misclassified by the target model out of the total number of generated AEs/UAEs. In addition, we introduce four metrics to evaluate the image quality of the generated AEs/UAEs. FID [50], SSIM [51], PSNR [52], and LPIPS [53] are used to measure the distribution distance between the generated and real images, the similarity of the image structures, the peak signal-to-noise ratio of the images, and the similarity of human visual perception, respectively.

**Implementation Details**. DeDiAttack uses SDM [26] as its framework. The DDIM sampler [20] is used for inversion and denoising. The number of sampling steps for inversion and the guidance scale are set to 5 and 0, respectively. The number of sampling steps for denoising and the guidance scale are set to 20 and 3, respectively. The optimization process for the latent representation uses the AdamW [54] optimizer with a learning rate set to 0.01. The number of optimization iterations for the attack is set to 20. The total number of deformable samples N is set to 5, and the update step size ρ is set to 1. For other hyperparameters of the loss function, we set α=10, β=100, and γ=100.

**Fairness of Comparison.** We note that restricted and unrestricted attacks are designed under substantially different perturbation constraints, optimization spaces, and computational settings. Therefore, our comparison is not intended to claim strict equivalence under an identical perturbation budget. Instead, it is used to show the practical trade-off between attack transferability and visual naturalness across the two dominant attack paradigms. For restricted attacks, we follow the standard pixel-space setting with a fixed perturbation budget. For unrestricted attacks, we use the officially recommended or commonly adopted settings of the corresponding methods. Under the same surrogate-target evaluation protocol, this comparison remains informative for assessing whether DeDiAttack can achieve competitive or superior transferability while preserving the natural appearance expected from unrestricted attacks.

### 4.2. Attacks on Normally Trained Models

Table 1 reports the attack performance of ten different attacks on normally trained models. DeDiAttack achieves the best attack performance on both CNN- and Transformer-based models. For example, DeDiAttack achieves an ASR of 99.2% in white-box tests on Res-50. In black-box tests, ViT-B results show that DeDiAttack improves the average ASR by approximately 7.2% compared to the second-best attack. The performance improvement is attributed to the fact that DeDiAttack performs deformation-based input transformation in the diffusion latent space, rather than directly injecting surrogate-specific high-frequency noise in pixel space. The elastic deformation mechanism enriches local geometric diversity and encourages the surrogate model to rely on more stable and architecture-agnostic features, thereby effectively improving the attack performance of the generated UAEs. Furthermore, the distribution alignment mechanism eliminates off-manifold invalid noise that would otherwise overfit the surrogate model. Together with the diffusion manifold prior, this alignment enables DeDiAttack to exhibit excellent transferability when facing unknown models.

### 4.3. Attacks on Defense Approaches

Table 2 reports the performance of the three most transferable attacks from Table 1 under different defense approaches. DeDiAttack exhibits strong robustness across various defense models. For example, DeDiAttack achieves ASRs 6.3% and 4.3% higher than the second-best attack when facing Bit-Red and DiffPure, respectively. The strong robustness benefits from the effective constraint of the distribution alignment mechanism. The mechanism forces the distribution of adversarial latent representations to strictly approximate the standard normal distribution, thereby eliminating invalid noise. The robust performance of DeDiAttack in defensive environments verifies the effectiveness of eliminating invalid noise for resisting model defenses.

### 4.4. Image Quality and Time Analysis

Table 3 reports the image quality and running time of the three most transferable attacks from Table 1. DeDiAttack achieves the best overall balance between attack performance, perceptual quality, and computational efficiency. Benefiting from the manifold prior knowledge of DMs and the structure preservation mechanism, DeDiAttack achieves optimal visual naturalness while maintaining high computational efficiency. For example, DeDiAttack achieves an FID and LPIPS score of 53.2 and 0.1465, respectively. The alignment operation of self-attention maps effectively suppresses structural distortions during the iterative optimization process. DeDiAttack utilizes the manifold prior knowledge of DMs to translate elastic deformations into smooth fluid changes, thereby eliminating unnatural artifacts.

### 4.5. Qualitative Comparison

**Figure 3:**
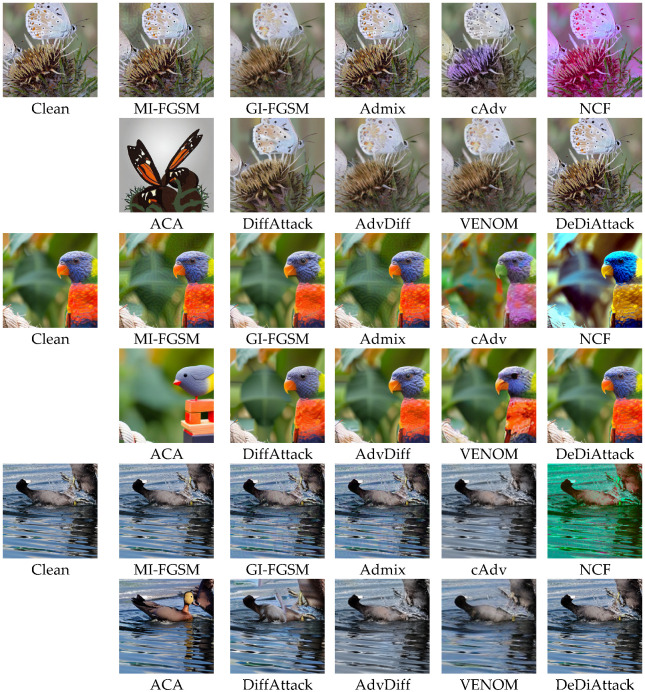
Visual comparison of AEs/UAEs generated by different attacks on Res-50.

Figure 3 displays the qualitative visual comparison results of AEs/UAEs generated by different attack methods. The UAEs generated by DeDiAttack exhibit outstanding visual quality. We observe that for restricted transfer attacks, AEs generated by MI-FGSM, GI-FGSM, and Admix exhibit significant pixel artifacts. For unrestricted attacks, UAEs generated by cAdv and NCF show obvious color distortions. UAEs generated by the ACA method exhibit visible structural deformations. UAEs generated by DiffAttack, AdvDiff, and VENOM show unreasonable distortions in minor details. In contrast, the visual effects of UAEs generated by DeDiAttack are highly natural and remain almost identical to the original images.

### 4.6. Ablation Study

Table 4 reports the ablation study results of the key mechanisms in DeDiAttack. We evaluate the impacts of elastic deformation, latent manifold perturbation, distribution alignment, and structure preservation mechanisms on the attack performance.

We first investigate the impact of the elastic deformation mechanism on the attack performance. The first row of Table 4 removes both elastic deformation and latent manifold perturbation. The absence of spatial deformations and classification loss significantly decreases the average ASR. The second row of Table 4 introduces the elastic deformation mechanism without constraining the deformation. The unconstrained elastic deformation produces excessive image distortions and increases FID by 2.7. The third row of Table 4 shows the experimental results after removing the elastic deformation mechanism. The lack of elastic deformation significantly reduces the transferability of the generated UAEs. The above results verify that the elastic deformation mechanism can enrich the details of generated UAEs and effectively improve their transferability. These results further indicate that elastic deformation is not merely an auxiliary augmentation operation; instead, it enriches transferable local details and helps the attack to move away from surrogate-specific solutions.

We subsequently investigate the impact of the distribution alignment mechanism on the attack performance. The fourth row of Table 4 shows the experimental results obtained when removing the distribution alignment mechanism. Unrestricted adversarial perturbations destroy the standard normal distribution of the data in the latent space. Distribution shifts degrade the injected perturbations into invalid noise during the denoising process of DMs. The lack of distribution alignment constraints reduces the transferability of generated UAEs and causes the average ASR to drop by 2.7%.

We finally investigate the impact of the structure preservation mechanism on image quality. The fifth row of Table 4 evaluates the influence of the structure preservation mechanism. Adversarial optimization in the latent space inevitably changes the content structure of generated images. Failing to constrain the self-attention maps during the diffusion denoising process severely damages the content structure of adversarial examples, while introducing the structure preservation mechanism helps to generate visually natural UAEs and reduces FID by approximately 6.3. This observation further supports that self-attention alignment is important for maintaining structural consistency during latent-space adversarial optimization.

**Figure 4:**
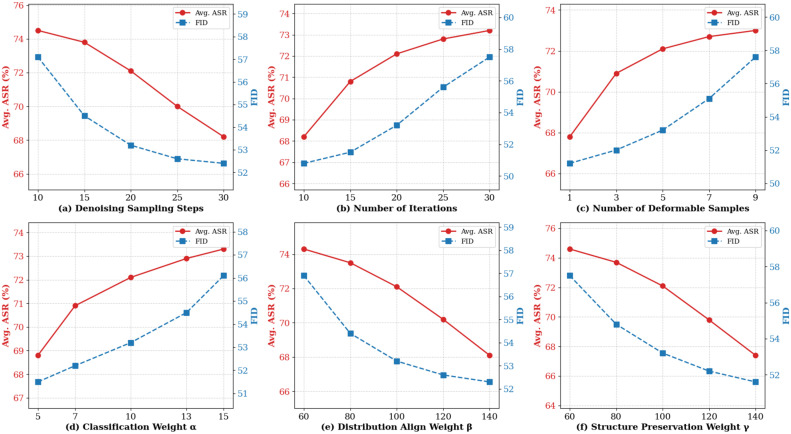
Hyperparameter ablation study results on UAEs generated on Res-50. (**a**). Denoising Sampling Steps (**b**). Number of Iterations (**c**). Number of Deformation Samples (**d**). Classification Weight α (**e**). Distribution Align Weight β (**f**). Structure Preservation Weight γ .

Figure 4 shows the ablation study results of six key hyperparameters in DeDiAttack. Each subfigure reports the impact of a specific parameter on the transferability and visual naturalness of the generated UAEs.

The denoising sampling steps directly affect the image reconstruction capabilities of the diffusion model. As shown in Figure 4a, we set the number of denoising sampling steps to 20 to ensure that the diffusion model reconstructs high-quality image details. Figure 4b shows the impact of the number of adversarial optimization iterations on the performance of generated UAEs. As increasing the number of iterations improves the average ASR but gradually exacerbates image distortion, we set the number of iterations to 20 to achieve a balance between transferability and image quality. Figure 4c evaluates the specific impact of the number of deformable samples. As increasing the number of samples provides richer spatial diversity for the attack, we set the number of deformable samples to 5 to strictly control the deformation magnitude.

Figure 4d–f report the experimental results of the three loss function weight parameters. The classification loss weight directly affects the strength of the adversarial gradient. The distribution alignment and structure preservation loss weights determine the degree of invalid noise elimination and the preservation of image content. We set the classification loss weight α to 10, and both the distribution alignment loss weight β and the structure preservation loss weight γ were set to 100 to achieve an optimal balance between attack performance and visual naturalness.

## 5. Discussion

The proposed DeDiAttack generates highly natural UAEs with outstanding transferability. However, the computational efficiency of DeDiAttack is significantly lower than that of traditional attacks such as PGD and MI-FGSM. Traditional attacks directly calculate adversarial perturbations through simple optimizations in the pixel space. Conversely, DeDiAttack relies on the complex denoising process of DMs. The iterative sampling mechanism introduces substantial computational overhead. The heavy computational burden restricts the practical deployment of DeDiAttack in strict real-time systems or highly resource-constrained environments.

In addition to computational cost, DeDiAttack’s performance is related to the diffusion backbone and the quality of its latent manifold. Our method assumes that the employed diffusion model provides sufficiently smooth and semantically meaningful latent trajectories. Different diffusion backbones, inversion strategies, or denoising samplers may lead to different trade-offs between reconstruction fidelity, deformation smoothness, and adversarial effectiveness. Therefore, the transferability gain of DeDiAttack may vary when the underlying generative backbone is changed.

The current study mainly focuses on image classification under the black-box transfer setting. Extending DeDiAttack to more complex tasks, such as object detection, semantic segmentation, multimodal perception, or more challenging sensing scenarios, still requires further investigation. In these settings, the interaction between deformation constraints, latent-space optimization, and task-specific decision boundaries may become more complicated.

From the perspective of sensor applications, DeDiAttack can also serve as a practical robustness analysis tool for vision-based sensing systems. In real-world scenarios, camera-based sensors, intelligent surveillance devices, autonomous perception platforms, and other imaging sensors often rely on deep neural networks for downstream recognition and decision-making. Evaluating whether these systems are vulnerable to highly natural and transferable adversarial examples is important for understanding their security boundaries. Because DeDiAttack generates adversarial samples with both high transferability and strong visual naturalness, it can be used to simulate more realistic threat patterns for testing the robustness of sensor data processing pipelines and vision-oriented sensing models in complex environments.

## 6. Conclusions

In this paper, we propose DeDiAttack to improve the cross-model transferability of UAEs. DeDiAttack utilizes the manifold prior knowledge of DMs to translate applied elastic deformations into smooth fluid changes. To prevent surrogate model overfitting and further boost attack performance, we introduce a distribution alignment mechanism that effectively eliminates invalid noise. Furthermore, a self-attention map alignment mechanism ensures the structural integrity of UAEs. Extensive experiments demonstrate that DeDiAttack generates highly natural UAEs, significantly outperforms existing attacks in cross-model transferability across diverse architectures, and exhibits strong robustness against various defense mechanisms. Our proposed method provides a powerful evaluation tool for assessing and enhancing the security of deep learning-based sensing and imaging systems, including camera-based sensors, visual perception modules, and image signal processing pipelines, thereby supporting more robust sensing-oriented visual recognition in practical environments.

## Figures and Tables

**Table 1:** Transferability comparison on normally trained models. The attack success rate (ASR, %) of each method is reported. For white-box attacks (where the surrogate model is identical to the target model), the background is set to gray. “Avg. ASR (%)” denotes the average ASR across all models. The best result is highlighted in bold, and the second-best result is underlined.

Surrogate Model	Attacks	Target Models						Avg. ASR (%)
		CNNs			Transformers			
		Res-50	VGG-19	Mob-v2	ViT-B	Swin-B	DeiT-B	
-	Clean	7.0	11.0	11.2	5.8	4.5	5.8	7.6
Res-50	MI-FGSM [8]	99.9	53.6	55.6	13.6	14.3	10.0	41.2
	GI-FGSM [9]	**100.0**	67.4	69.2	19.2	22.4	13.3	48.6
	Admix [10]	**100.0**	75.8	78.6	27.2	31.0	29.2	57.0
	cAdv [14]	97.3	36.9	43.7	28.2	21.4	25.1	42.1
	NCF [15]	89.9	72.2	71.7	37.9	26.8	29.3	54.6
	ACA [17]	90.0	68.3	70.0	51.9	51.3	52.0	63.9
	DiffAttack [18]	96.3	75.6	77.1	51.2	56.2	50.1	67.8
	AdvDiff [36]	98.8	65.8	69.5	57.9	58.5	60.6	68.5
	VENOM [37]	98.7	62.5	63.2	41.2	45.7	40.6	58.7
	DeDiAttack	99.2	**77.4**	**79.5**	**58.3**	**61.0**	**57.3**	**72.1**
Mob-v2	MI-FGSM [8]	56.4	54.7	99.8	14.2	13.1	10.9	41.5
	GI-FGSM [9]	70.5	66.8	**100.0**	20.4	21.3	14.8	49.0
	Admix [10]	79.5	76.9	**100.0**	28.5	32.1	28.1	57.5
	cAdv [14]	37.2	39.5	96.3	27.8	19.7	23.7	40.7
	NCF [15]	65.6	72.3	92.5	33.9	25.1	24.8	52.4
	ACA [17]	60.5	64.4	93.3	49.0	47.2	49.1	60.6
	DiffAttack [18]	75.7	76.8	97.9	47.7	55.4	47.0	66.8
	AdvDiff [36]	64.8	63.2	98.7	55.6	56.2	58.2	66.1
	VENOM [37]	68.6	68.2	98.5	40.6	46.8	38.6	60.2
	DeDiAttack	**73.4**	**75.7**	99.0	**60.1**	**60.6**	**58.7**	**71.3**
ViT-B	MI-FGSM [8]	27.5	28.6	29.1	99.7	37.8	38.9	43.6
	GI-FGSM [9]	36.8	37.5	38.1	**100.0**	46.9	47.9	51.2
	Admix [10]	46.5	47.2	47.9	99.8	56.6	57.8	59.3
	cAdv [14]	33.7	33.8	40.7	97.7	30.3	49.0	47.5
	NCF [15]	55.1	60.1	61.6	72.6	32.8	35.1	52.9
	ACA [17]	62.7	64.1	66.9	81.2	57.4	65.1	66.2
	DiffAttack [18]	59.9	59.1	63.2	95.3	69.7	77.2	70.7
	AdvDiff [36]	60.5	59.7	63.8	96.3	70.4	**78.0**	71.4
	VENOM [37]	48.6	54.8	48.2	96.5	59.9	70.3	63.1
	DeDiAttack	**74.6**	**73.3**	**75.6**	96.9	**74.9**	76.5	**78.6**

**Table 2:** ASR (%) comparison of UAEs generated on Res-50 by the three most transferable attacks from Table 1 under various defense approaches. The best result is highlighted in bold, and the second-best result is underlined.

	HGD	R&P	JPEG	Bit-Red	DiffPure
ACA [17]	31.3	30.1	35.9	35.5	38.9
DiffAttack [18]	37.9	36.7	40.5	43.7	50.5
AdvDiff [36]	45.4	46.8	45.2	46.6	53.2
DeDiAttack	**49.3**	**48.6**	**50.9**	**52.9**	**57.5**

**Table 3:** Comparison of image quality and running time of UAEs generated on Res-50 by the three most transferable attacks from Table 1. The best result is highlighted in bold, and the second-best result is underlined. “↓” indicates that the smaller the evaluation metric, the better; “↑” indicates that the larger the evaluation metric, the better.

Attacks	TIME (s) ↓	Avg.ASR (%) ↑	FID ↓	SSIM ↑	PSNR ↑	LPIPS ↓
ACA [17]	125.3	63.9	**45.6**	0.5137	18.09	0.3361
DiffAttack [18]	29.9	67.8	62.6	0.7365	**22.66**	0.1478
AdvDiff [36]	20.5	68.5	58.1	0.2658	12.84	0.6983
DeDiAttack	**19.8**	**72.1**	53.2	**0.7496**	22.05	**0.1465**

**Table 4:** Ablation studies of various mechanisms in the proposed method based on the average ASR (%) and FID of UAEs generated on Res-50. “↓” indicates that the smaller the evaluation metric, the better; “↑” indicates that the larger the evaluation metric, the better.

Elastic Deformation	Latent Manifold Perturbation	Distribution Alignment	Structure Preservation	Avg.ASR (%) ↑	FID ↓
		√	√	64.5	48.8
√		√	√	67.2	51.5
	√	√	√	67.1	50.2
√	√		√	69.4	52.7
√	√	√		74.6	59.5
√	√	√	√	72.1	53.2

## Data Availability

Publicly available datasets are analyzed in this study. These data can be found at https://github.com/cleverhans-lab/cleverhans/tree/master/cleverhans_v3.1.0/examples/nips17_adversarial_competition/dataset (accessed on 26 April 2026). The custom datasets generated during the study are available from the corresponding author upon reasonable request.

## Abbreviations

The following abbreviations are used in this manuscript:

DNNs	Deep Neural Networks
AEs	Adversarial Examples
UAEs	Unrestricted Adversarial Examples
DMs	Diffusion Models
SDM	Stable Diffusion Model
ASR	Attack Success Rate
